# The effect of exercise referral schemes and self-management strategies on use of prescription analgesics among community-dwelling older adults: registry linkage with randomised controlled trials

**DOI:** 10.1186/s12877-024-05235-3

**Published:** 2024-07-31

**Authors:** Nanna Herning Svensson, Jonas Bloch Thorlund, Pia Øllgaard Olsen, Jens Søndergaard, Sonja Wehberg, Helene Støttrup Andersen, Paolo Caserotti, Trine Thilsing

**Affiliations:** 1 Research Unit of General Practice, Department of Public Health, University of Southern Denmark, Campusvej 55, Odense M, 5230 Denmark; 2 Research Unit for Musculoskeletal Function and Physiotherapy, Department of Sports Science and Clinical Biomechanics, University of Southern Denmark, Campusvej 55, Odense M, 5230 Denmark; 3 Department of Health, Culture and Development, Municipality of Tønder, Wegners plads 2, Tønder, 6270 Denmark; 4 Centre for Active and Healthy Aging, Department of Sports Science and Clinical Biomechanics, University of Southern Denmark, Campusvej 55, Odense M, 5230 Denmark

## Abstract

**Background and objective:**

Exercise referral schemes and self-management strategies have shown positive effects on patient-reported and objectively measured outcomes, such as increased functional capacity and physical activity level. However, the impact of these interventions on analgesic use remains uncertain. We hypothesised that exercise referral schemes, either utilised alone or in combination with self-management strategies, is more effective in reducing use of prescription analgesics compared with a self-management strategy only.

**Subjects and methods:**

We utilised data from two completed randomised controlled trials, namely *The Welfare Innovation in Primary Prevention* (*n* = 121) and *The SITLESS project* (*n* = 338), and information from the national Danish health registries, including the National Prescription Registry. The two trials have investigated the effectiveness of interventions, which include exercise referral schemes and self-management strategies, on various aspects such as physical function and levels of physical activity among community-dwelling older adults. The studies were conducted in the period 2015–2020 and comprised older adults aged 65+ years, living in three different Danish municipalities. Participants were recruited through nationally regulated preventive home-visits. To estimate changes in use of prescription analgesics over time, a linear fixed effects regression model was applied. The outcome measure was the mean total yearly defined daily dose of analgesics.

**Results:**

All intervention groups showed a within-group increase in overall analgesic use, though not statistically significantly different from zero. There were no differences in estimated changes in mean total yearly defined daily dose when comparing the intervention groups to the group receiving the least extensive intervention (self-management strategies/control). The findings indicated that exercise referral schemes and self-management strategies, whether administrated individually or in combination, did not result in a reduction in analgesic use over time.

**Supplementary Information:**

The online version contains supplementary material available at 10.1186/s12877-024-05235-3.

## Introduction

Analgesic use is common among older adults, aged 65 years and above, with an estimated prevalence of prescribed analgesics of 21% and 29% in Danish older men and women, respectively [1]. The high proportion is partly explained by a high prevalence of chronic pain in this age group [2]. Use of analgesics is associated with an elevated risk of experiencing both side effects and adverse events. For instance, opioid use can lead to side effects such as constipation, nausea, and dizziness [3]. On the other hand, non-steroidal anti-inflammatory medication (NSAIDs) may result in epigastric pain, nausea, and diarrhoea [4]. The most significant adverse event associated with consumption of paracetamol is unintentional overdosing, which can cause acute liver failure [5]. Besides, increased age is associated with higher risk of polypharmacy leading in turn to an increased risk of hospital admissions [6]. Therefore, identifying effective approaches without negative side effects which may alleviate pain and subsequently reduce the use of analgesics among older adults, should be regarded as an important healthcare concern for both health authorities and older individuals.

Physical activity is considered a vital element in retaining health and normal physiologic function during the life span [7], and several studies have shown positive effects on pain severity [2, 7, 8]. Exercise referral schemes (ERS) are nationally regulated interventions in several countries. The main purpose of ERS is to serve as a primary care strategy to enhance physical activity at an individual level. ERS can be defined as a tailored program consisting of structured exercise, recommended by a primary healthcare professional. These customised exercise programmes incorporate ongoing supervision and monitoring, and are usually implemented within the community settings, like public recreational facilities, and facilitated by the local municipalities [9, 10]. A potential way to enhance the effectiveness of ERS is to combine them with self-management strategies (SMS), which involves behaviour changes techniques to increase exercise adherence and promote adoption of physically active behaviours. The concept of *self-management* is typically associated with an individual’s capacity to handle chronic diseases. Consequently, the patient’s proactive involvement is a fundamental element within the self-management framework [11]. Lorig et al. highlights five principal self-management concepts yet notes that many studies incorporating SMS do not encompass all five concepts. These key concepts encompass (a) problem-oriented approaches, (b) decision-making processes, (c) assistance in identifying and utilising relevant resources (d) establishing a collaborative relationship between the individual and healthcare provider, and (e) initiating action and making progress [11]. Previous studies have found that non-pharmacologic cognitive-behavioural therapy is associated with small to moderate efficacy for chronic or subacute lower back pain [12–14]. In addition, a two-armed randomised controlled trial (RCT) that included individuals aged 18–75 years showed no between-group differences in pain intensity at 6–12 months follow-up, when comparing individuals randomised to cognitive functional therapy or group-based exercise. However, both intervention groups experienced a drop in pain intensity from baseline to follow-up [15].

While ERS and SMS alone may decrease pain levels, it is currently unknown whether these interventions may be capable of reducing the use of prescription analgesic among community-dwelling older adults, particularly when the interventions do not specifically target pain or analgesic use. We hypothesised that ERS, either utilised alone or in combination with self-management strategies, is more effective in reducing use of prescription analgesics compared with SMS only. This study aimed to investigate the effect of ERS alone or in combination with SMS, on use of analgesics in Danish community-dwelling older adults.

## Methods

In this study we used data from two completed RCTs, *The Welfare Innovation in Primary Prevention* (WIPP) (ClinicalTrials.gov ID: NCT04531852) [16] and *The SITLESS project* (SITLESS) (ClinicalTrials.gov ID: NCT02629666) [17]. Both studies, designed as complex interventions combining ERS and SMS, had the primary aim of modifying behaviours associated with premature risk of functional decline and disability in community-dwelling older adults. WIPP aimed at increasing physical function, quality of life, and healthy life years, and SITLESS aimed at determining whether the effects of ERS were enhanced by adding on an SMS-program to reduce sedentary behaviour, increase physical activity, and improve health and quality of life. The primary result from the WIPP study revealed that the combined intervention (ERS + SMS) significantly improved physical function compared to SMS alone [16], which underscores the efficacy of the combined intervention. Both WIPP and SITLESS were international multicentre studies, but this study includes only Danish participants. The studies were conducted in the years 2015–2020 in the three Danish municipalities; Esbjerg (WIPP), Slagelse (WIPP), and Odense (SITLESS). For a detailed overview of the inclusion criteria in WIPP and SITLESS see Additional file 1. Data from these studies were linked on an individual level with national Danish health registries, including the National Prescription Registry.

### Study population

**Fig. 1:**
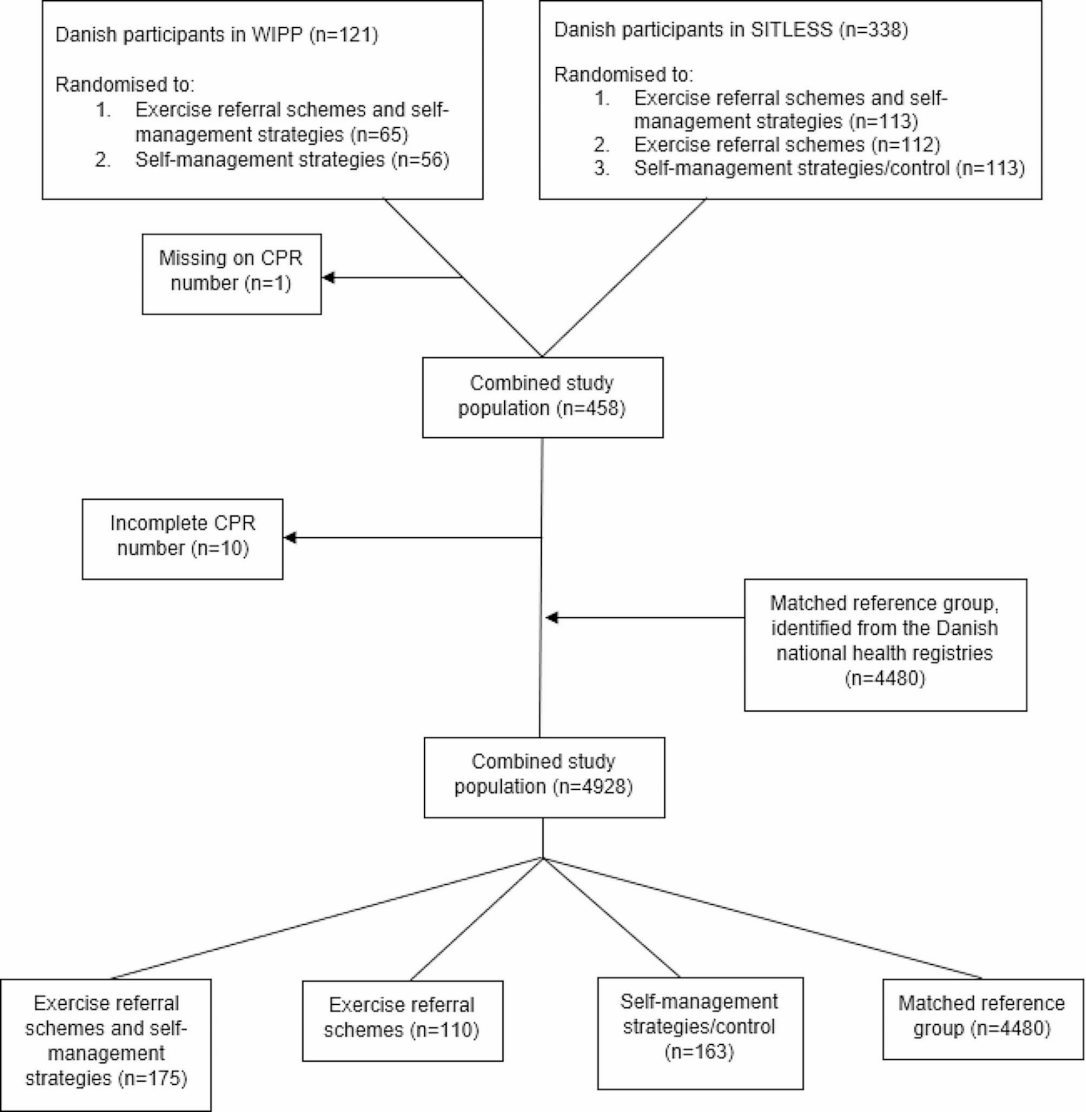
Flowchart of the study population

Participants from both WIPP and SITLESS were community-dwelling older adults recruited through the Danish nationally regulated preventive home-visits offered by the municipalities. According to Danish regulations, the Act on Social Services § 79a, preventive home-visits are offered to self-reliant individuals (i.e., who do not receive municipal home care services on a regular basis) aged 75+ years, or 65+ years if evaluated as vulnerable [18, 19]. Inclusion criteria for this study were (I) included and randomised to an intervention in either WIPP or SITLESS, and (II) complete social security number (CPR number). All Danish citizens are assigned with a CPR number, a unique personal identifier in the Danish health registries. To see the study population flow from the WIPP and SITLESS studies see Fig. 1.

A reference group from the general Danish population were identified from the national registries and matched on age and sex, and vital status (alive) at index date in a 1:10 ratio.

### Interventions

In short, WIPP was a two-armed RCT, where individuals were randomised to either an ERS + SMS or SMS intervention [16]. SITLESS was a three-armed RCT with following intervention groups: ERS + SMS, ERS, and SMS/control [17]. In SITLESS a control group was included, however this group received recommendations on physical activity and was invited to two seminars focusing on (1) health-enhancing benefit of increasing physical activity, and reducing sedentary behaviour, and (2) healthy nutrition. Participants were encouraged to ask questions and discuss their experiences with both themes [17]. Based on this, we refer to the group as having received an SMS/control intervention, despite differences in the structured and theory-based SMS-programmes with respect to the volume and intensity of targeted behaviour-change strategies. See Table 1 for description of the interventions in WIPP and SITLESS.

**Table 1:** Description of the interventions in WIPP and SITLESS

	WIPP*	SITLESS**
**Interventions** ERS: Exercise referral schemes SMS: Self-management strategies	**ERS + SMS**: The ERS intervention consisted of structured training sessions (resistance, balance, and aerobic exercises) at a 1-hour duration twice a week for 12 weeks. This was combined with an SMS intervention (see below), consisting of 8 group sessions each at a 1.5-hours duration during a 24-weeks period [16]. **SMS**: The SMS intervention was based on participant-centred activities, health information, and motivational conversations, to encourage the participant to believe in their own ability to act and thereby reinforce them to undertake a healthier behaviour. The SMS focused on physical activity, sedentary behaviour, nutrition, incontinence, and few other risk factors for functional loss. Beyond that, they tested new behaviour strategies in practice, such as visits to the local training facility. The SMS intervention was conducted in groups of 6–12 individuals, with 12 sessions, each at a 1.5-hours duration, during a period of 24 weeks [16].	**ERS + SMS**: The intervention was a combination of the ERS, consisting of supervised training (for 16 weeks) and an SMS intervention consisting of 6 group sessions, one 1-to-1 session and four telephone calls, lasting 30 weeks in total. The duration of the ERS was 1 hour per session whereas the duration of the SMS varied between 20–60 min. per session. The SMS intervention was conducted by the same specialists who were responsible for the supervised training in the ERS group. The aim of the SMS intervention was for the participants to set a realistic long-term goal to increase physical activity and decrease sedentary behaviour, as well as self-monitoring including pedometer and activity diary [17]. **ERS**: The physical intervention consisted of two supervised training sessions pr. week for 16 weeks, focusing on aerobic training, strength, and endurance exercises, as well as flexibility and balance exercises [17]. **Control group receiving optimised usual care (referred to as SMS/control)**: The group received flyers on WHOs recommendations for physical activity and two seminars. The seminars had a 2-hour duration and focused on (1) physical activity, sedentary behaviour, and exercise and (2) effects of healthy nutrition in older adults. Beyond that, the group received Christmas cards and were tested at 4 different time points (pre-intervention, post-intervention, at 12 months follow-up and at 18 months follow-up) [17].

* The first intervention session in WIPP was on 24 September 2018, and the last session was on 9 August 2019

** The first intervention session in SITLESS was on 3 November 2016, and the last session was on 16 November 2017

In this study, we combined the groups from WIPP and SITLESS leading to three groups: the combined ERS + SMS, ERS, and SMS/control.

### Data sources

Individual level data from WIPP and SITTLESS was combined with data from the Danish National Prescription Registry (DNPR) [20], The Population Register at Statistics Denmark [21], the Danish Register of Cause of Death [22], the register concerning immigration and emigration at Statistics Denmark [23], and the Danish National Patient Registry [24].

### Outcomes

#### Main outcome

The main outcome measure was the mean number of defined daily dose (DDD) of analgesics consumed per year according to redeemed prescriptions in the DNPR covering the Anatomical Therapeutic Chemical (ATC) codes N02BE01 (paracetamol), M01A* (non-steroidal anti-inflammatory drugs - NSAIDs), and N02A* (opioids) [see Additional file 2]. The inclusion of the three categories of analgesics is based on the most consumed analgesics in Denmark [25, 26]. A DDD is assigned to every drug substance by the WHO and represents the average maintenance dose per day in an adult for a drug that is used for its original indication [27]. Only oral and rectal routes of administration for opioids were included. For each redeemed prescription, the amount of DDD, as provided in the DNPR, was calculated by dividing the milligrams of the drug substance per one unit (e.g. on tablet) by the DDD for that specific drug. Subsequently, the DDD for each drug was multiplied by the number of packages. Baseline analgesic use was defined as the utilisation of analgesics during the 12-months period leading up to the index date [day  -361 to -1]. The index date of each participant in the intervention group, as well as their matched controls in the reference group, was defined as the date of the first ERS + SMS, ERS or SMS/control session. To ensure equal length of periods for all participants, we define the baseline period of 360 days and the follow-up period of 720 days. The index date [day 0] was included in the follow-up period. In case of death or migration, time at risk were less than two years. As the follow-up period was two years [day 0 to 720], we divided the total amount of DDD in the follow-up period by two as well as considering risk time, to estimate the mean total yearly DDD.

#### Secondary outcomes

We estimated the mean total yearly DDD for paracetamol and NSAIDs separately. For opioids we calculated the total amount of morphine milligram equivalent (MME) per individual per year. To derive MME, the DDD was calculated by accounting for the number of packages and afterwards multiplied with the current opioid dose (mg/DDD) multiplied by an equianalgesic ratio [28]. For conversion table [see Additional file 2].

### Statistics

Baseline descriptive statistics were reported as counts (n) and proportions (%) and the included variables are described in [Additional file 3]. An intention-to-treat approach was used which implies that all individuals who were randomised to an intervention in either WIPP or SITLESS were included in the analyses, and analysed according to the intervention group to which they were assigned. To compare the difference in estimated within-group changes in mean total yearly DDD from baseline to follow-up in the ERS + SMS, ERS, and matched reference group compared to the SMS/control group we applied a linear fixed effects regression model accounting for clustering in matched groups. In a fixed effect model, one cannot estimate the effect of covariates which are constant. The SMS/control group was used as the reference group in the analyses as it was the group receiving the least extensive intervention. The relevant regression coefficient was modelled as an interaction term between time and intervention group (time x group). To estimate the use of analgesics over time in 6-months intervals, we estimated the mean level of DDD in each 6-months interval. Only individuals alive during the entire 6-months period were included in the analyses. The level of statistical significance was set at 5%. All statistical analyses were performed in Stata/BE 17.0 (StataCorp LLC, 2019).

## Results

A total of 448 participants from WIPP and SITLESS were included in the analyses. Of the included 61% (*n* = 273) were women and the majority (62%, *n* = 278) were in the age group 75–84 years (Table 2). 44% of the total intervention group redeemed no prescriptions of the analgesic types included in this study during the baseline period [Additional file 4]. The descriptive statistics stratified by project can be found in [Additional file 5].

**Table 2:** Baseline characteristics of the study population

		SMS/control	ERS + SMS	ERS	Total intervention group	Matched reference group
**Total**	N (%)	163 (100)	175 (100)	110 (100)	448 (100)	4480 (100)
**Register data**						
**Sex**	Women	99 (61)	108 (62)	66 (60)	273 (61)	2730 (61)
	Men	64 (39)	67 (38)	44 (40)	175 (39)	1750 (39)
**Age group**	65–74 years	37 (23)	34 (19)	30 (27)	101 (23)	1010 (23)
	75–84 years	95 (58)	118 (67)	65 (59)	278 (62)	2780 (62)
	85–94 years	31 (19)	23 (13)	15 (14)	69 (15)	690 (15)
**Marital status**	Married/registered partnership	56 (34)	64 (37)	49 (45)	169 (38)	2291 (51)
	Widowed/divorced/ not married	107 (66)	111 (63)	61 (55)	279 (62)	2189 (49)
**Cancer status at baseline**	Cancer at baseline	10 (6)	16 (9)	7 (6)	33 (7)	259 (6)
**Project data**						
**Project**	SITLESS	110 (67)	113 (65)	110 (100)	333 (74)	-
	WIPP	53 (33)	62 (35)	-	115 (26)	-
	Matched reference group	-	-	-	-	4480 (10)
**Body Mass Index (BMI)**	BMI < 25.0	46 (28)	50 (29)	39 (35)	135 (30)	-
	BMI ≥ 25.0*	117 (72)	125 (71)	71 (65)	313 (70)	-
	Missing	-	-	-	-	4480 (100)

The baseline characteristics of the study population are given in counts and percentages [N (%)] by intervention group comprising of exercise referral schemes (ERS) and self-management strategies (SMS/control), managed either alone or in combination

*Missings less than 5 (n = < 5) have been included under the majority “BMI ≥ 25.0”.

### Main outcome - overall analgesic use

**Fig. 2:**
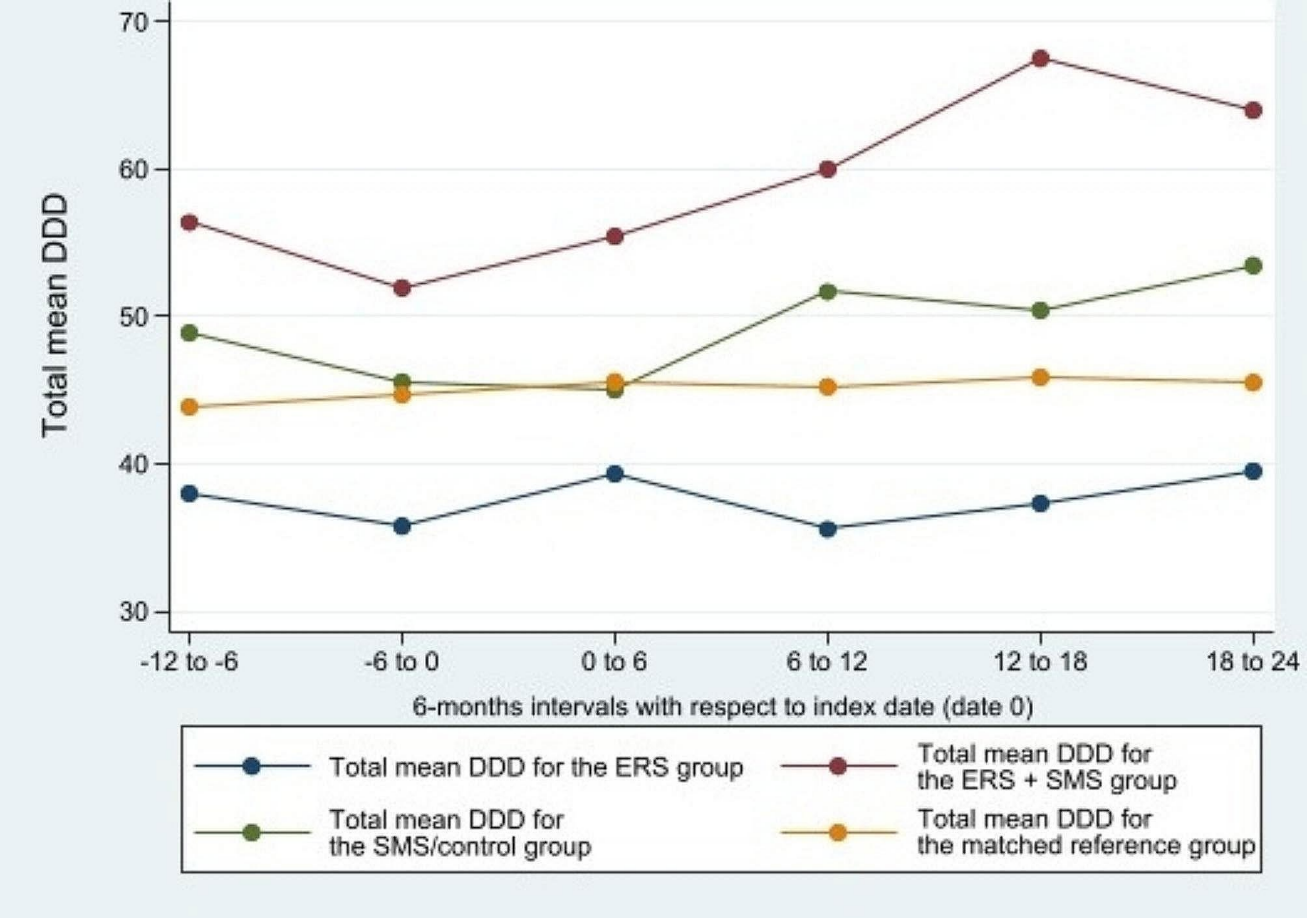
Simple illustration of average overall analgesic use over time

Results for both main and secondary analyses are presented in Table 3. No statistically significant within-group changes in overall analgesic use, from baseline to follow-up, were found in any of the three intervention groups. The estimated within-group changes ranged from 1.5 mean DDD per year (95% CI: -14.1 ; 17.1) in the ERS group to 38.3 (95% CI: -5.1 ; 81.7) in the ERS + SMS group. Only the matched reference group showed a statistically significant within-group increase in mean use of total yearly DDD from baseline to follow-up on 9.4 (95% CI: 6.6 ; 12.3). No statistically significant between-group differences were observed when comparing the intervention groups and the matched reference group to the SMS/control group. Figure 2 illustrates total mean DDD of overall analgesic use over time, displayed in 6-months intervals, for the intervention groups including the matched reference group.

**Table 3:** Results for main and secondary analyses

	Baseline	Follow-up	Estimated within-group changes (95% CI)	Difference in estimated changes versus the SMS/control group (95% CI)**
**Main outcome** **(group x time interactions)**: **Total yearly DDD of overall use of analgesic** ^**#**^	**Mean** ^≠^ **(SD)**	**Mean (SD)**		
SMS/control	94.5 (144.1)	106.5 (148.0)	12.0 (-3.4 ; 27.4)	
ERS	74.4 (116.1)	75.9 (120.3)	1.5 (-14.1 ; 17.1)	-10.5 (-32.4 ; 11.4)
ERS + SMS	108.6 (177.4)	146.9 (346.2)	38.3 (-5.1 ; 81.7)	26.3 (-19.8 ; 72.3)
Matched reference group	88.7 (162.4)	98.2 (160.0)	9.4 (6.6 ; 12.3)	-2.6 (-18.3 ; 13.1)
**Secondary outcomes** **(group x time interactions)**: **Total yearly DDD/MME** ^¶^				
**Paracetamol** **(N02BE01)**	**Mean (SD)**	**Mean (SD)**		
SMS/control	70.6 (112.5)	84.1 (123.5)	13.5 (1.3 ; 25.7)	
ERS	61.9 (100.8)	62.8 (104.8)	0.9 (-12.1 ; 13.9)	-12.6 (-30.4 ; 5.3)
ERS + SMS	79.5 (123.1)	116.5 (320.1)	37.1 (-5.0 ; 79.2)	23.6 (-20.2 ; 67.4)
Matched reference group	63.7 (115.0)	75.3 (120.5)	11.5 (9.3 ; 13.8)	-2.0 (-14.3 ; 10.4)
**NSAIDs** (M01A*)	**Mean (SD)**	**Mean (SD)**		
SMS/control	13.3 (52.2)	7.9 (24.0)	-5.4 (-10.9 ; 0.1)	
ERS	7.8 (33.9)	7.3 (32.5)	-0.6 (-3.8 ; 2.7)	4.9 (-1.5 ; 11.2)
ERS + SMS	15.5 (66.8)	13.9 (55.3)	-1.6 (-11.3 ; 8.1)	3.8 (-7.3 ; 14.9)
Matched reference group	12.7 (53.7)	10.4 (43.9)	-2.3 (-3.6 ; -1.1)	3.1 (-2.6 ; 8.8)
**Opioids** (N02A*)	**Mean MME (SD)**	**Mean MME (SD)**		
SMS/control	741.2 (3281.0)	1077.0 (3386.4)	335.8 (-96.0 ; 767.7)	
ERS	367.0 (1610.1)	493.1 (1832.2)	126.1 (-131.5 ; 383.7)	-209.7 (-712.6 ; 293.1)
ERS + SMS	836.3 (3558.5)	1032.7 (4349.8)	196.5 (-152.7 ; 545.6)	-139.4 (-694.7 ; 416.0)
Matched reference group	898.1 (4049.0)	969.1 (4136.0)	71.0 (5.7 ; 136.2)	-264.9 (-699.5 ; 169.7)

Effectiveness of exercise referral schemes (ERS) and self-management strategies (SMS/control) on mean total yearly Defined Daily Dose (DDD) of analgesics* among community-dwelling older adults who have been randomised to either SMS/control (*n* = 163), ERS (*n* = 110), or ERS + SMS (*n* = 175). Matched reference group consisted of *n* = 4480

* Analgesics cover N02BE01 (paracetamol), M01A* (NSAIDs), and N02A* (opioids). Combination products with codeine (N02AJ*) and glucosamine (M01AX05) were excluded

** Results from the linear fixed effects regression model accounting for clustering

# Overall use of analgesics covers use of N02BE01 (paracetamol), M01A* (NSAIDs), and N02A* (opioids)

≠ Mean is based on the individuals’ use of analgesics and their risk time in the given period. Individuals who have not received any analgesics are still included in the calculation of mean

¶ Morphine milligram equivalents (MME) are given only for opioids

The figure displays mean values of total Defined Daily Dose (DDD) over time within groups in 6-months intervals in relation to the index date (date 0) for individuals alive in each assessment point. To get an overview of the data used to generate the figure [see Additional file 6].

### Secondary outcomes – stratified per analgesic type

There was a statistically significant increase in use of paracetamol and opioids, and a statistically significant decrease in use of NSAIDs within the matched reference group from baseline to follow-up. Among the intervention groups, the SMS/control group showed a statistically significant within-group increase in mean paracetamol use of 13.5 DDD per year (95% CI: 1.3 ; 25.7). The remaining results among the intervention groups were not statistically significant. Illustrations of total mean DDD/MME in specific analgesic drug use over time, displayed in 6-months intervals, can be found in [Additional file 7].

## Discussion

We observed an apparent increase in the use of overall prescription analgesics within the three intervention groups, though none reached statistical significance. Increases over time were statistically significant solely within the matched reference group and among the SMS/control group when specifically focusing on consumption of paracetamol. We found no statistically significant difference between the groups receiving ERS + SMS or ERS when compared to the SMS/control group. Thus, it does not seem likely that a more comprehensive intervention to promote physically active and healthy lifestyle among community-dwelling older adults has any influence on analgesic use.

To our knowledge this is the first study investigating whether complex interventions combining ERS + SMS have an impact on use of redeemed prescriptions for analgesics among community-dwelling older adults. Previous research within this field has focused primarily on patient-reported and objectively measured outcomes such as functional capacity, physiological measures, pain intensity, and quality of life [15, 16, 29].

The main aims of the WIPP and SITLESS interventions were to enhance physical activity levels, reduce sedentary behaviour, and improve overall quality of life besides additional health outcomes. However, the interventions did not contain specific elements targeting analgesic use, such as review of medication lists, initiating the possibility of ceasing medication in collaboration with a general practitioner, etc. This may be one of the reasons for the observed results. Most of the participants in the study were 75+ years and analgesic use was followed over a two-year period. Health declines at a faster rate with increasing age [30], which often translates into a higher consumption of analgesics [1]. Accordingly, we found a statistically significant within-group increase in overall analgesic use in the matched reference group. When comparing the within-group changes in overall use of analgesics, among the intervention groups with the reference group matched on sex and age, it showed that the matched reference group had an increase in mean use of total yearly DDD that was close to the increase observed in the SMS/control group (Table 3). In the matched reference group, we found an increased use of paracetamol and opioids, opposite to a reduced use of NSAIDs. The reduced use of NSAIDs among this group is a positive finding and align with findings from another Danish study [31]. This as prescriptions of NSAIDs is not recommended to older adults, due to an elevated risk of adverse events [32]. The rise in utilisation of opioids among older individuals is undesirable. Particularly, given the heightened attention to the opioid epidemic, efforts should be directed towards reducing the consumption of this specific type of medication. A previous study aimed at investigating changes in use of opioids after participating in a standardised treatment program for osteoarthritis (Good Life with osteoArthritis in Denmark - GLA: D), found no effect of such program among patients with knee or hip osteoarthritis and chronic opioid use [28]. The GLA: D study, like WIPP and SITLESS, comprise exercise therapy and behaviour changes techniques, but none of the studies specifically aimed at reducing analgesic use [16, 17, 33]. Another study using data from the Good Life with osteoArthritis in Denmark (GLA: D) reported that the proportion of analgesic users among older patients with knee or hip osteoarthritis decreased over time, when participating in the GLA: D exercise interventions [34]. Nevertheless, individual patients may experience onset of pain or increased pain severity from being more physical active [35], which could lead to use of pain medication. A systematic review focusing on patient enablers and barriers of deprescribing finds that the patients’ feeling of improvement while taking the medication and lack of time and support from the general practitioner was perceived as barriers to cease medication. Conversely, positive influence including a good relationship with the general practitioner was considered an enabler towards deprescribing. In addition, lack of symptoms, the feeling of not needing the medication, and a slowly reduction in the dose of medication combined with knowing the possibility to return to the medication if the patient perceived symptoms returning, increased the openness of deprescribing [36]. This pinpoints that cessation of medication is complex and interventions aiming at deprescribing analgesics should acknowledge the importance of including the patients’ general practitioner, as a core element in the deprescribing process.

### Limitations

Although the present study used high quality data from already completed RCTs and the national Danish health registries some limitations should also be acknowledged. We do not have information about the use of over-the-counter (OTC) medication. Both Paracetamol and NSAIDs are available OTC at a relatively low price in Denmark. We acknowledge that some participants may have used OTC analgesics. However, frequent use of OTC analgesics is not inexpensive and in Denmark there is a financial incentive to use prescribed medication as most of it is reimbursed and covered by universal healthcare [20]. As most Danes (85%) aged 65 years and above receive prescribed medication [37], we believe that frequently used analgesics are most likely prescribed and only a limited amount is purchased OTC. Considering this, we believe that the limitation related to the lack of information on OTC medication is not significant in our study. Even though the Danish registry data is of high quality, is it worth mentioning, that the utilisation of registry data for prescription medication does not provide insights into whether the individuals consume their prescribed medication or whether they share medication with others.

We pooled data from two RCTs which requires that study populations are similar. While the studies had similar inclusion criteria such as community-dwelling older adults with sedentary behaviour for extended periods, there were variations in the inclusion criteria regarding the level of functional capacity. A limitation, regarding the use of data from the two RCTs, that may have impacted the statistical power is the absence of data from one of the municipalities participating in the WIPP study. This occurred because the municipality opted not to provide us with the requested data, which they are entitled to do under Danish legislation. The broad confidence intervals in some of the analyses in this study may suggest a lack of statistical power, potentially compromising the detection of effects over time. A way to increase the statistical power would be to increase the sample size, yet this was not an option in this study, as we used data from two already completed RCTs.

In our study, we employed a mean-based statistical analysis approach. However, we acknowledge that alternative approaches, such as logistic regression or median regression models, could have been utilised. The mean approach is sensible to outliers. Given that only a small subset of participants exhibited high usage of prescription analgesics, which positioned them as outliers, we opted for the mean-based approach. This approach also provides a more illustrative representation of the data.

Both the WIPP and SITLESS studies relied on voluntary participation, resulting in a self-selected group of individuals, which is why we included a matched reference group. While self-selection introduces a potential bias that is challenging to avoid, it is worth noting that the study participants were community-dwelling older adults with varying levels of functional loss and disability risks and the recruitment was performed by municipal staff through the Danish nationally regulated preventive home-visits. Therefore, we believe the findings of this study are to some extent generalisable to the average community-dwelling older adults aged 65 years and above in Denmark.

### Conclusion and further perspective

This study found that complex interventions comprising exercise referral schemes, either utilised alone or in combination with self-management strategies, did not reduce the overall use of prescription analgesics among Danish community-dwelling older adults over time. In future projects offering similar interventions aiming at improving overall health in older adults, it would be interesting to include focus on analgesic use. In addition, future studies should investigate whether different levels of exercise might mediate the association between exercise, perceived pain, and use of analgesics among older adults.

### Electronic supplementary material

Below is the link to the electronic supplementary material.

Supplementary Material 1

Supplementary Material 2

Supplementary Material 3

Supplementary Material 4

Supplementary Material 5

Supplementary Material 6

Supplementary Material 7

## Data Availability

The datasets supporting the conclusion of this article are not publicly available due to privacy and confidentiality restrictions pertaining to person-level health information. However, metadata such as names on variables used from the registries as well as the analytic codes used to perform the analyses are available from the corresponding author on reasonable request.
