# Supplementary material for: The effect of exercise referral schemes and self-management strategies on use of prescription analgesics among community-dwelling older adults: registry linkage with randomised controlled trials
Source: BMC Geriatr. 2024 Jul 31;24:641. doi: 10.1186/s12877-024-05235-3 (PMC11293001; doi:10.1186/s12877-024-05235-3)
Supplement: Supplementary file 1 — Supplementary Material 1 [file 12877_2024_5235_MOESM1_ESM.docx]

# Additional file 1

**Inclusion criteria in the WIPP and SITLESS studies**

The purpose of WIPP was to increase physical function by either ERS and SMS, or SMS among participants who are at risk of functional loss and disability [1]. In the SITLESS study, the purpose was to determine whether ERS could be enhanced by SMS to reduce sedentary behaviour, increase physical activity, and improve health and quality of life among insufficiently active older adults aged 65+ years [2].

|  | WIPP | SITLESS |
| --- | --- | --- |
| Inclusion criteria | Participants were included if they fulfilled at least one of the following risk factors:   - Low functional capacity with a Short Physical Performance Battery (SPPB) score ≤ 9. - Fatigability score ≥ 15 based on The Pittsburgh Fatigability Scale. - Being physically active no more than once a week and more than 8 hours daily sitting time. - Experienced more than two fall episodes during the past 12 months.      - Pain score on ≥ 20 based on The Brief Pain Inventory. | Participants were eligible if they fulfilled the following criteria:   - 65+ years. - Community-dwelling. - Able to walk without help from another for at least 2 min or without a walking aid. - No major physical limitations, shown by a Short Physical Performance Battery (SPPB) score ≥ 4. - Insufficient level of activity determined by the question “*Do you perform regular physical activity for at least 30 minutes five or more days of the week (referring only to physical activity that makes the participant become out of breath while doing it or such that it doesn’t allow him/her to maintain a conversation while doing the activity) (do not count regular walking)”*. - Sedentary behaviour for long periods of time, determined by the question *“For most days, do you feel you sit for too long (6-8 hours or more a day)?”*. |
| Exclusion criteria | Participants were excluded if they had:   - SPPB score >10 irrespective of whether they met any other inclusion criteria. | Participants were excluded if one of the following criteria were fulfilled:   - Moderate to severe dementia, using a cut-off of three using the six-item screener [3] to identify cognitive impairment. - A medical condition that may interfere with the study design. - Unstable medical conditions (e.g., uncontrolled hypertension) or symptomatic cardiovascular diseases which contraindicates participation in physical activity. - Expect not being able to attend 75% of the ERS sessions. - Participated in an ERS intervention within the past six months. |

**References**

1. Olsen P, Tully MA, Del Pozo Cruz B, et al.; Community-based exercise enhanced by a self-management programme to promote independent living in older adults: a pragmatic randomised controlled trial. *Age Ageing* 2022;**51**(7). doi: 10.1093/ageing/afac137.

2. Giné-Garriga M, Coll-Planas L, Guerra M, et al.; The SITLESS project: exercise referral schemes enhanced by self-management strategies to battle sedentary behaviour in older adults: study protocol for a randomised controlled trial. *Trials* 2017;**18**(1):221. doi: 10.1186/s13063-017-1956-x.

3. Callahan CM, Unverzagt FW, Hui SL, et al.; Six-item screener to identify cognitive impairment among potential subjects for clinical research. *Med Care* 2002;**40**(9):771-81. doi: 10.1097/00005650-200209000-00007.
