# Supplementary material for: The effect of exercise referral schemes and self-management strategies on use of prescription analgesics among community-dwelling older adults: registry linkage with randomised controlled trials
Source: BMC Geriatr. 2024 Jul 31;24:641. doi: 10.1186/s12877-024-05235-3 (PMC11293001; doi:10.1186/s12877-024-05235-3)
Supplement: Supplementary file 2 — Supplementary Material 2 [file 12877_2024_5235_MOESM2_ESM.docx]

# Additional file 2

**Overview of included Anatomical Therapeutic Chemical (ATC) codes, analgesic class, mg/DDD, equianalgesic ratio, and morphine milligram equivalent (MME) consumed by the study participants during the study period**

Combination products with codeine (N02AJ*) and Glucosamine (M01AX05) were excluded, the latter because the Danish Health Authorities does not perceive it as an NSAID, as it has another mechanism of action [1].

The included analgesics cover the following ATC codes: N02BE01 (paracetamol), M01A* (non-steroidal anti-inflammatory drugs - NSAIDs), and N02A* (opioids). The inclusion of the three categories of analgesics is based on the most consumed analgesics in Denmark [2, 3].

| **ATC** | **Analgesic class** | **Drug** | **Administrative route*** | **mg/DDD** | **Equianalgesic ratio (opioids only)** | **MME** |
| --- | --- | --- | --- | --- | --- | --- |
| N02BE01 | Paracetamol | Paracetamol | O and R | 3000 |  |  |
|  |  |  |  |  |  |  |
| M01AB01 | NSAID | Indometacin | O and R | 100 |  |  |
| M01AB05 | NSAID | Diclofenac | O and R | 100 |  |  |
| M01AB08 | NSAID | Etodolac | O | 400 |  |  |
| M01AB55 | NSAID | Diclofenac, combinations | O | 100 |  |  |
| M01AC01 | NSAID | Piroxicam | O | 20 |  |  |
| M01AC05 | NSAID | Iornoxicam | O | 12 |  |  |
| M01AE01 | NSAID | Ibuprofen | O | 1200 |  |  |
| M01AE02 | NSAID | Naproxen | O and R | 500 |  |  |
| M01AE11 | NSAID | Tiaprofenic acid | O | 600 |  |  |
| M01AE14 | NSAID | Dexibuprofen | O | 800 |  |  |
| M01AG02 | NSAID | Tolfenamic acid | O | 300 |  |  |
| M01AH01 | NSAID | Celecoxib | O | 200 |  |  |
| M01AX01 | NSAID | Nabumetone | O | 1000 |  |  |
|  |  |  |  |  |  |  |
| N02AA01 | Opioid | Morphine | O | 100 | 1.0 | 100 |
| N02AA05 | Opioid | Oxycodone | O | 75 | 1.5 | 112.5 |
| N02AB02 | Opioid | Pethidine | O and R | 400 | 0.1 | 40 |
| N02AG02 | Opioid | Ketobemidone and antispasmodics | O and R | 25 [O] /  50 [R] | 2.0 [O] /  3.0 [R] | 50 [O] /  150 [R] |
| N02AX02 | Opioid | Tramadol | O and R | 300 | 0.2 | 60 |
| N02AX06 | Opioid | Tapentadol | O | 400 | 0.4 | 160 |

* Administrative route; O – oral, R – rectal.

**References**

1. The Danish Health Data Authority. *Analgesic drug* <https://sundhedsdatastyrelsen.dk/da/find-tal-og-analyser/tal-og-analyser/laegemidler/smertestillende-medicin> (Date Accessed 2022 Accessed, date last accessed)

2. Burghle A, Pottegård A, Rasmussen M, et al.; Use of analgesics in Denmark: A national survey. *Basic Clin Pharmacol Toxicol* 2023;**132**(4):321-327. doi: 10.1111/bcpt.13837.

3. Nissen SK, Pottegård A, Ryg J; Trends of Opioid Utilisation in Denmark: A Nationwide Study. *Drugs Real World Outcomes* 2019;**6**(4):155-164. doi: 10.1007/s40801-019-00163-w.
