# Supplementary material for: The effect of exercise referral schemes and self-management strategies on use of prescription analgesics among community-dwelling older adults: registry linkage with randomised controlled trials
Source: BMC Geriatr. 2024 Jul 31;24:641. doi: 10.1186/s12877-024-05235-3 (PMC11293001; doi:10.1186/s12877-024-05235-3)
Supplement: Supplementary file 3 — Supplementary Material 3 [file 12877_2024_5235_MOESM3_ESM.docx]

# Additional file 3

**Variables included in the descriptive statistics**

| **Variable** | **Description and categorisation of variable** |
| --- | --- |
| **Register data** |  |
| Sex | Categorical:   - Women - Men   Derived from The Population Register at Statistics Denmark. |
| Age group | Categorical:   - 65-74 years - 75-84 years - 85-94 years   Derived from The Population Register at Statistics Denmark. |
| Marital status | Categorical:   - Widowed/divorced/not married - Married/registered partnership   Derived from The Population Register at Statistics Denmark. |
| Cancer status at baseline | Categorical:   - Cancer at baseline - No cancer at baseline   Data concerning cancer status are derived through The Danish National Patient Register, and the variable ‘cancer at baseline’ is measured as occurrence of any cancer type, except for dermatological cancer, two years prior to index date.  Due to transition in the reporting systems at the hospitals we have data up to and including the 31^st^ of December 2018. 57 participants from WIPP have not complete baseline registration. However, 24 of the 57 participants have an index date in January 2019 and therefore less than a month lack of baseline registration on cancer status. The participants with the latest index date are in august 2019. |
| **Project data** |  |
| Study | Categorical:   - WIPP - SITLESS - Matched reference group   Derived from the two studies. |
| Body Mass Index (BMI) | Categorical:   - Underweight to normal weight (BMI <18.5–24.9) - Overweight (BMI ≥25.0) - Missing   If missing were less than five (n= <5) then it was included in the majority, in this case ‘Underweight to normal weight (BMI <18.5-24.9)’ |
