# Supplementary material for: The effect of exercise referral schemes and self-management strategies on use of prescription analgesics among community-dwelling older adults: registry linkage with randomised controlled trials
Source: BMC Geriatr. 2024 Jul 31;24:641. doi: 10.1186/s12877-024-05235-3 (PMC11293001; doi:10.1186/s12877-024-05235-3)
Supplement: Supplementary file 4 — Supplementary Material 4 [file 12877_2024_5235_MOESM4_ESM.docx]

# Additional file 4

**Distribution of participants on the redeemed prescription categories N02BE01, M01A*, and N02A** at baseline by intervention groups comprising of exercise referral schemes (ERS) and/or self-management strategies (SMS)**

|  | |  | **SMS/control** | **ERS + SMS** | **ERS** | **Total intervention group** | **Matched reference group** |
| --- | --- | --- | --- | --- | --- | --- | --- |
| **Total** | N (%) | | 163 (100) | 175 (100) | 110 (100) | 448 (100) | 4480 (100) |
|  |  | |  |  |  |  |  |
| **Total number of overall redeemed prescriptions** | 0 prescriptions | | 73 (45) | 74 (42) | 50 (45) | 197 (44) | 2250 (50) |
|  | 1 prescription | | 25 (15) | 28 (16) | 19 (17) | 72 (16) | 545 (12) |
|  | 2 prescriptions | | 13 (8) | 20 (11) | 11 (10) | 44 (10) | 369 (8) |
|  | 3-10 prescriptions | | 41 (25) | 39 (22) | 25 (23) | 105 (23) | 947 (21) |
|  | 10+ prescriptions | | 11 (7) | 14 (8) | 5 (5) | 30 (7) | 369 (8) |
|  |  | |  |  |  |  |  |
| **Paracetamol: Total number of redeemed prescriptions** | 0 prescriptions | | 80 (49) | 87 (50) | 58 (53) | 225 (50) | 2511 (56) |
|  | 1 prescription | | 27 (17) | 25 (14) | 20 (18) | 72 (16) | 638 (14) |
|  | 2 prescriptions | | 14 (9) | 18 (10) | 8 (7) | 40 (9) | 359 (8) |
|  | 3-6 prescriptions | | 34 (21) | 28 (16) | 18 (16) | 80 (18) | 612 (14) |
|  | +6 prescriptions | | 8 (5) | 17 (10) | 6 (5) | 31 (7) | 360 (8) |
|  |  | |  |  |  |  |  |
| **NSAIDs: Total number of redeemed prescriptions** | 0 prescriptions | | 137 (84) | 143 (82) | 94 (85) | 374 (83) | 3808 (85) |
|  | 1 prescription | | 14 (9) | 20 (11) | - | 46 (10) | 351 (8) |
|  | +1 prescription | | - | - | 16 (15)*** | - | - |
|  | +2 prescriptions | | 12 (7) | 12 (7) | - | 28 (6) | 321 (7) |
|  |  | |  |  |  |  |  |
| **Opioids: Total number of redeemed prescriptions** | 0 prescriptions | | 139 (85) | 147 (84) | 92 (84) | 378 (84) | 3765 (84) |
|  | 1-2 prescriptions | | 13 (8) | 12 (7) | 13 (12) | 38 (8) | 289 (6) |
|  | +2 prescriptions | | 11 (7) | 16 (9) | 5 (5) | 32 (7) | 426 (10) |

* Glucosamine (M01AX05) was excluded because the Danish Health Authorities do not perceive it as an NSAID.

** Combination products with codeine were excluded.

*** Due to a low number of observations (n<5) a +1-prescription category were included.
