# Supplementary material for: The effect of exercise referral schemes and self-management strategies on use of prescription analgesics among community-dwelling older adults: registry linkage with randomised controlled trials
Source: BMC Geriatr. 2024 Jul 31;24:641. doi: 10.1186/s12877-024-05235-3 (PMC11293001; doi:10.1186/s12877-024-05235-3)
Supplement: Supplementary file 5 — Supplementary Material 5 [file 12877_2024_5235_MOESM5_ESM.docx]

# Additional file 5

**Baseline characteristics of the study population stratified by project (WIPP and SITLESS)**

Given in counts and percentages [N (%)] by intervention groups comprising of exercise referral schemes (ERS) and/or self-management strategies (SMS/control).

**SITLESS WIPP**

|  |  | | **SMS/control** | **ERS + SMS** | **ERS** | **Matched reference group** | **SMS/control** | **ERS + SMS** | **Matched reference group** | **Total intervention group** |
| --- | --- | --- | --- | --- | --- | --- | --- | --- | --- | --- |
| **Total** | |  | 110 (100) | 113 (100) | 110 (100) | 3330 (100) | 53 (100) | 62 (100) | 1150 (100) | 448 (100) |
|  | |  |  |  |  |  |  |  |  |  |
| **Register data** | |  |  |  |  |  |  |  |  |  |
|  | |  |  |  |  |  |  |  |  |  |
| **Sex** | | Women | 65 (59) | 65 (58) | 66 (60) | 1960 (59) | 34 (64) | 43 (69) | 770 (67) | 273 (61) |
|  | | Men | 45 (41) | 48 (42) | 44 (40) | 1370 (41) | 19 (36) | 19 (31) | 380 (33) | 175 (39) |
|  | |  |  |  |  |  |  |  |  |  |
| **Age group** | | 65-74 years | 29 (26) | 24 (21) | 30 (27) | 830 (25) | 8 (15) | 10 (16) | 180 (16) | 101 (23) |
|  | | 75-84 years | 65 (59) | 79 (70) | 65 (59) | 2090 (63) | 30 (57) | 39 (63) | 690 (60) | 278 (62) |
|  | | 85-94 years | 16 (15) | 10 (9) | 15 (14) | 410 (12) | 15 (28) | 13 (21) | 280 (24) | 69 (15) |
|  | |  |  |  |  |  |  |  |  |  |
| **Marital status** | | Married/registered partnership | 45 (41) | 50 (44) | 49 (45) | 1750 (53) | 11 (21) | 14 (23) | 541 (47) | 169 (38) |
|  | | Widowed/divorced/not married | 65 (59) | 63 (56) | 61 (55) | 1580 (47) | 42 (79) | 48 (77) | 609 (53) | 279 (62) |
|  | |  |  |  |  |  |  |  |  |  |
| **Project data*** | |  |  |  |  |  |  |  |  |  |
|  | |  |  |  |  |  |  |  |  |  |
| **Project** | | SITLESS | - | - | - | - | - | - | - | 333 (74) |
|  | | WIPP | - | - | - | - | - | - | - | 115 (26) |
|  | | Matched reference group | - | - | - | - | - | - | - | - |
|  | |  |  |  |  |  |  |  |  |  |
| **BMI categories** | | Underweight and normal weight (BMI <18.5-24.9) | 31 (28) | 37 (33) | 39 (35) | - | 15 (28) | 13 (21) | - | 135 (30) |
|  | | Overweight (BMI ≥25) | 79 (72) | 76 (67) | 71 (65) | - | 38 (72) | 49 (79) | - | 313 (70) |
|  | | Missing | - | - | - | 3330 (100) | - | - | 1150 (100) | - |

***** Information about cancer at baseline is excluded in the stratified analyses due to low numbers of observations (n<5).
