# Supplementary material for: The effect of exercise referral schemes and self-management strategies on use of prescription analgesics among community-dwelling older adults: registry linkage with randomised controlled trials
Source: BMC Geriatr. 2024 Jul 31;24:641. doi: 10.1186/s12877-024-05235-3 (PMC11293001; doi:10.1186/s12877-024-05235-3)
Supplement: Supplementary file 6 — Supplementary Material 6 [file 12877_2024_5235_MOESM6_ESM.docx]

# Additional file 6

**Data used to generate Figure 2 with 6-months intervals for the main outcome overall analgesic use of paracetamol, NSAIDs, and opioids**

The table includes 6-months intervals, total amount of Defined Daily Dose (DDD), number of participants, mean DDD, standard deviation (SD), 10^th^ (p10), 50^th^ (p50), and 90^th^ (p90) percentile.

| **ERS + SMS** | | | | | | | |
| --- | --- | --- | --- | --- | --- | --- | --- |
| **Month** | **Total DDD** | **Participants** | **Mean DDD** | **SD** | **p10** | **p50** | **p90** |
| -12 to -6 | 9868 | 175 | 56 | 93 | 0 | 0 | 167 |
| -6 to 0 | 9095 | 175 | 52 | 93 | 0 | 0 | 200 |
| 0 to 6 | 9702 | 175 | 55 | 96 | 0 | 0 | 187 |
| 6 to 12 | 10446 | 174 | 60 | 93 | 0 | 17 | 192 |
| 12 to 18 | 11607 | 172 | 67 | 99 | 0 | 17 | 220 |
| 18 to 24 | 10878 | 170 | 64 | 103 | 0 | 10 | 205 |
| **ERS** | | | | | | | |
| **Month** | **Total DDD** | **Participants** | **Mean DDD** | **SD** | **p10** | **p50** | **p90** |
| -12 to -6 | 4180 | 110 | 38 | 62 | 0 | 0 | 108 |
| -6 to 0 | 3932 | 110 | 36 | 59 | 0 | 0 | 105 |
| 0 to 6 | 4326 | 110 | 39 | 64 | 0 | 0 | 148 |
| 6 to 12 | 3876 | 109 | 36 | 67 | 0 | 0 | 110 |
| 12 to 18 | 4068 | 109 | 37 | 63 | 0 | 0 | 120 |
| 18 to 24 | 4269 | 108 | 40 | 65 | 0 | 0 | 150 |
| **SMS/CONTROL** | | | | | | | |
| **Month** | **Total DDD** | **Participants** | **Mean DDD** | **SD** | **p10** | **p50** | **p90** |
| -12 to -6 | 7969 | 163 | 49 | 79 | 0 | 0 | 183 |
| -6 to 0 | 7430 | 163 | 46 | 73 | 0 | 0 | 150 |
| 0 to 6 | 7339 | 163 | 45 | 72 | 0 | 0 | 150 |
| 6 to 12 | 8217 | 159 | 52 | 76 | 0 | 17 | 178 |
| 12 to 18 | 7918 | 157 | 50 | 78 | 0 | 0 | 177 |
| 18 to 24 | 8293 | 155 | 54 | 85 | 0 | 0 | 206 |
| **MATCHED REFERENCE GROUP** | | | | | | | |
| **Month** | **Total DDD** | **Participants** | **Mean DDD** | **SD** | **p10** | **p50** | **p90** |
| -12 to -6 | 196571 | 4480 | 44 | 86 | 0 | 0 | 150 |
| -6 to 0 | 200174 | 4480 | 45 | 83 | 0 | 0 | 150 |
| 0 to 6 | 204299 | 4480 | 46 | 83 | 0 | 0 | 155 |
| 6 to 12 | 197643 | 4366 | 45 | 82 | 0 | 0 | 156 |
| 12 to 18 | 195116 | 4253 | 46 | 82 | 0 | 0 | 154 |
| 18 to 24 | 187794 | 4128 | 45 | 80 | 0 | 0 | 157 |
