# Supplementary material for: The effect of exercise referral schemes and self-management strategies on use of prescription analgesics among community-dwelling older adults: registry linkage with randomised controlled trials
Source: BMC Geriatr. 2024 Jul 31;24:641. doi: 10.1186/s12877-024-05235-3 (PMC11293001; doi:10.1186/s12877-024-05235-3)
Supplement: Supplementary file 7 — Supplementary Material 7 [file 12877_2024_5235_MOESM7_ESM.docx]

# Additional file 7

## Illustrations of total mean DDD/MME in specific analgesic drug use over time, displayed in 6-months intervals, only for those alive in each assessment point.

**Total mean Defined Daily Dose of paracetamol over time**

Mean values within groups are displayed in 6-months intervals in relation to the index date (date 0). Including a table holding data used to generate the figure. The table includes 6-months intervals, total amount of DDD, number of participants, mean DDD, standard deviation (SD), 10^th^ (p10), 50^th^ (p50), and 90^th^ (p90) percentile.

**
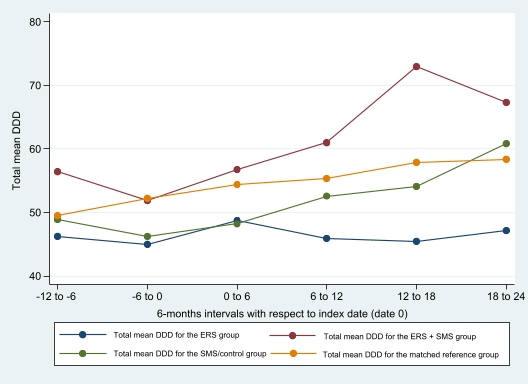
**

| **ERS + SMS** | | | | | | | |
| --- | --- | --- | --- | --- | --- | --- | --- |
| **Month** | **Total DDD** | **Participants** | **Mean DDD** | **SD** | **p10** | **p50** | **p90** |
| -12 to -6 | 7219 | 128 | 56 | 71 | 0 | 50 | 150 |
| -6 to 0 | 6639 | 128 | 52 | 69 | 0 | 17 | 155 |
| 0 to 6 | 7265 | 128 | 57 | 71 | 0 | 33 | 177 |
| 6 to 12 | 7741 | 127 | 61 | 75 | 0 | 50 | 177 |
| 12 to 18 | 9118 | 125 | 73 | 76 | 0 | 50 | 177 |
| 18 to 24 | 8283 | 123 | 67 | 75 | 0 | 50 | 177 |
| **ERS** | | | | | | | |
| **Month** | **Total DDD** | **Participants** | **Mean DDD** | **SD** | **p10** | **p50** | **p90** |
| -12 to -6 | 3424 | 74 | 46 | 57 | 0 | 28 | 100 |
| -6 to 0 | 3332 | 74 | 45 | 60 | 0 | 8 | 150 |
| 0 to 6 | 3607 | 74 | 49 | 61 | 0 | 50 | 150 |
| 6 to 12 | 3354 | 73 | 46 | 67 | 0 | 17 | 177 |
| 12 to 18 | 3320 | 73 | 45 | 61 | 0 | 22 | 133 |
| 18 to 24 | 3395 | 72 | 47 | 62 | 0 | 18 | 150 |
| **SMS/CONTROL** | | | | | | | |
| **Month** | **Total DDD** | **Participants** | **Mean DDD** | **SD** | **p10** | **p50** | **p90** |
| -12 to -6 | 5917 | 121 | 49 | 67 | 0 | 17 | 150 |
| -6 to 0 | 5596 | 121 | 46 | 62 | 0 | 17 | 133 |
| 0 to 6 | 5833 | 121 | 48 | 67 | 0 | 17 | 150 |
| 6 to 12 | 6206 | 118 | 53 | 65 | 0 | 22 | 150 |
| 12 to 18 | 6277 | 116 | 54 | 71 | 0 | 20 | 150 |
| 18 to 24 | 6933 | 114 | 61 | 79 | 0 | 22 | 200 |
| **MATCHED REFERENCE GROUP** | | | | | | | |
| **Month** | **Total DDD** | **Participants** | **Mean DDD** | **SD** | **p10** | **p50** | **p90** |
| -12 to -6 | 138473 | 2799 | 49 | 69 | 0 | 17 | 150 |
| -6 to 0 | 146281 | 2799 | 52 | 70 | 0 | 17 | 150 |
| 0 to 6 | 152266 | 2799 | 54 | 71 | 0 | 22 | 167 |
| 6 to 12 | 149818 | 2707 | 55 | 71 | 0 | 33 | 158 |
| 12 to 18 | 151653 | 2619 | 58 | 71 | 0 | 44 | 172 |
| 18 to 24 | 146694 | 2512 | 58 | 70 | 0 | 49 | 158 |

**Total mean Defined Daily Dose (DDD) of NSAIDs over time**

Mean values within groups are displayed in 6-months intervals in relation to the index date (date 0). Including a table holding data used to generate the figure. The table includes 6-months intervals, total amount of DDD, number of participants, mean DDD, standard deviation (SD), 10^th^ (p10), 50^th^ (p50), and 90^th^ (p90) percentile.

**
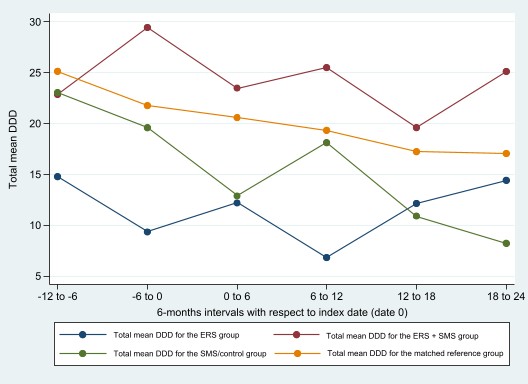
**

| **ERS + SMS** | | | | | | | |
| --- | --- | --- | --- | --- | --- | --- | --- |
| **Month** | **Total DDD** | **Participants** | **Mean DDD** | **SD** | **p10** | **p50** | **p90** |
| -12 to -6 | 1189 | 52 | 23 | 48 | 0 | 0 | 67 |
| -6 to 0 | 1532 | 52 | 29 | 76 | 0 | 0 | 67 |
| 0 to 6 | 1222 | 52 | 23 | 61 | 0 | 0 | 67 |
| 6 to 12 | 1326 | 52 | 25 | 43 | 0 | 2 | 67 |
| 12 to 18 | 1018 | 52 | 20 | 41 | 0 | 0 | 67 |
| 18 to 24 | 1307 | 52 | 25 | 62 | 0 | 0 | 90 |
| **ERS** | | | | | | | |
| **Month** | **Total DDD** | **Participants** | **Mean DDD** | **SD** | **p10** | **p50** | **p90** |
| -12 to -6 | 517 | 35 | 15 | 41 | 0 | 0 | 33 |
| -6 to 0 | 327 | 35 | 9 | 22 | 0 | 0 | 33 |
| 0 to 6 | 427 | 35 | 12 | 35 | 0 | 0 | 33 |
| 6 to 12 | 238 | 35 | 7 | 34 | 0 | 0 | 8 |
| 12 to 18 | 425 | 35 | 12 | 25 | 0 | 0 | 63 |
| 18 to 24 | 505 | 35 | 14 | 31 | 0 | 0 | 45 |
| **SMS/CONTROL** | | | | | | | |
| **Month** | **Total DDD** | **Participants** | **Mean DDD** | **SD** | **p10** | **p50** | **p90** |
| -12 to -6 | 1175 | 51 | 23 | 59 | 0 | 0 | 67 |
| -6 to 0 | 998 | 51 | 20 | 36 | 0 | 0 | 55 |
| 0 to 6 | 657 | 51 | 13 | 34 | 0 | 0 | 33 |
| 6 to 12 | 923 | 51 | 18 | 32 | 0 | 0 | 50 |
| 12 to 18 | 556 | 51 | 11 | 32 | 0 | 0 | 25 |
| 18 to 24 | 420 | 51 | 8 | 17 | 0 | 0 | 25 |
| **MATCHED REFERENCE GROUP** | | | | | | | |
| **Month** | **Total DDD** | **Participants** | **Mean DDD** | **SD** | **p10** | **p50** | **p90** |
| -12 to -6 | 30558 | 1215 | 25 | 55 | 0 | 0 | 90 |
| -6 to 0 | 26434 | 1215 | 22 | 48 | 0 | 0 | 75 |
| 0 to 6 | 25006 | 1215 | 21 | 45 | 0 | 0 | 67 |
| 6 to 12 | 23153 | 1197 | 19 | 45 | 0 | 0 | 67 |
| 12 to 18 | 20197 | 1173 | 17 | 42 | 0 | 0 | 57 |
| 18 to 24 | 19526 | 1145 | 17 | 42 | 0 | 0 | 50 |

**Total mean Morphine Milligram Equivalents (MME)** **of opioids over time**

Mean values within groups are displayed in 6-months intervals in relation to the index date (date 0). Including a table holding data used to generate the figure. The table includes 6-months intervals, total amount of MME, number of participants, mean MME, standard deviation (SD), 10^th^ (p10), 50^th^ (p50), and 90^th^ (p90) percentile.

**
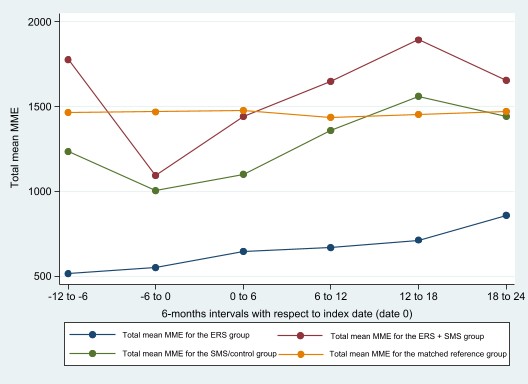
**

| **ERS + SMS** | | | | | | | |
| --- | --- | --- | --- | --- | --- | --- | --- |
| **Month** | **Total MME** | **Participants** | **Mean MME** | **SD** | **p10** | **p50** | **p90** |
| -12 to -6 | 90585 | 51 | 1776 | 3820 | 0 | 0 | 6000 |
| -6 to 0 | 55760 | 51 | 1093 | 2576 | 0 | 0 | 3000 |
| 0 to 6 | 73550 | 51 | 1442 | 3100 | 0 | 0 | 4000 |
| 6 to 12 | 84125 | 51 | 1650 | 4266 | 0 | 0 | 4000 |
| 12 to 18 | 96565 | 51 | 1893 | 3966 | 0 | 200 | 7920 |
| 18 to 24 | 81145 | 49 | 1656 | 3591 | 0 | 0 | 7925 |
| **ERS** | | | | | | | |
| **Month** | **Total MME** | **Participants** | **Mean MME** | **SD** | **p10** | **p50** | **p90** |
| -12 to -6 | 19515 | 38 | 514 | 1271 | 0 | 0 | 1750 |
| -6 to 0 | 20860 | 38 | 549 | 1410 | 0 | 0 | 2840 |
| 0 to 6 | 24535 | 38 | 646 | 1568 | 0 | 0 | 3000 |
| 6 to 12 | 24675 | 37 | 667 | 1586 | 0 | 0 | 1125 |
| 12 to 18 | 26375 | 37 | 713 | 1546 | 0 | 0 | 4000 |
| 18 to 24 | 30935 | 36 | 859 | 1985 | 0 | 0 | 5000 |
| **SMS/CONTROL** | | | | | | | |
| **Month** | **Total MME** | **Participants** | **Mean MME** | **SD** | **p10** | **p50** | **p90** |
| -12 to -6 | 66656 | 54 | 1234 | 3169 | 0 | 0 | 4000 |
| -6 to 0 | 54160 | 54 | 1003 | 2615 | 0 | 0 | 3810 |
| 0 to 6 | 59500 | 54 | 1102 | 2230 | 0 | 0 | 4000 |
| 6 to 12 | 69310 | 51 | 1359 | 3264 | 0 | 0 | 4000 |
| 12 to 18 | 76530 | 49 | 1562 | 2797 | 0 | 0 | 5000 |
| 18 to 24 | 67855 | 47 | 1444 | 2411 | 0 | 0 | 5000 |
| **MATCHED REFERENCE GROUP** | | | | | | | |
| **Month** | **Total MME** | **Participants** | **Mean MME** | **SD** | **p10** | **p50** | **p90** |
| -12 to -6 | 2007819 | 1370 | 1466 | 3536 | 0 | 0 | 5000 |
| -6 to 0 | 2011578 | 1370 | 1468 | 3559 | 0 | 0 | 5000 |
| 0 to 6 | 2023883 | 1370 | 1477 | 3472 | 0 | 0 | 5000 |
| 6 to 12 | 1864422 | 1297 | 1437 | 3361 | 0 | 0 | 5000 |
| 12 to 18 | 1782723 | 1226 | 1454 | 3546 | 0 | 0 | 5000 |
| 18 to 24 | 1680967 | 1142 | 1472 | 3392 | 0 | 0 | 5000 |
